# The New Danger of Thirdhand Smoke: Why Passive Smoking Does Not Stop at Secondhand Smoke

**DOI:** 10.1289/ehp.1103956

**Published:** 2011-10-01

**Authors:** Carmela Protano, Matteo Vitali

**Affiliations:** Department of Public Health and Infectious Diseases, Sapienza University, Rome, Italy, E-mail: matteo.vitali@uniroma1.it

Passive smoking exposure is a topic of great concern for public health because of its well-known adverse effects on human health (International Agency for Research on Cancer 2004). Two news articles on this topic were published in the February 2011 issue of *Environmental Health Perspectives* (Burton 2011; Lubick 2011). Lubick (2011) discussed the global health burden of secondhand smoke, and Burton (2011) emphasized a new and alarming consequence of  smoking in indoor environments—“thirdhand smoke”—a term first coined in 2006 (Szabo 2006).

Secondhand smoke is defined as “the combination of smoke emitted from the burning end of a cigarette or other tobacco products and smoke exhaled by the smoker” (World Health Organization 2007). Thus, secondhand smoke exposure consists of an unintentional inhalation of smoke that occurs close to people smoking and/or in indoor environments where tobacco was recently used.

Thirdhand smoke is a complex phenomenon resulting from residual tobacco smoke pollutants that adhere to the clothing and hair of smokers and to surfaces, furnishings, and dust in indoor environments. These pollutants persist long after the clearing of secondhand smoke. They are reemitted into the gas phase or react with oxidants or other compounds present in the environment to form secondary contaminants, some of which are carcinogenic or otherwise toxic for human health (Matt et al. 2011). Thus, thirdhand smoke exposure consists of unintentional intake (mainly through inhalation but also via ingestion and dermal routes) of tobacco smoke and other related chemicals that occurs in the absence of concurrent smoking. Exposure can even take place long after smoking has ceased, through close contact with smokers and in indoor environments in which tobacco is regularly smoked.

Lubick (2011) considers secondhand smoke synonymous with passive smoking, as do the majority of the authors publishing on this topic. However, in light of new evidence about thirdhand smoke (Matt et al. 2011), it is no longer appropriate to use the term “secondhand smoke” as a synonym for passive smoking or environmental tobacco smoke, because it represents a *pars pro toto*. In other words, using the term “secondhand smoke” mistakes one part of the problem for the whole. Instead, we propose that “passive smoking” or “environmental tobacco smoke” be used as a more inclusive term to describe any tobacco smoke exposure outside of active smoking.

This question of terminology is of particular concern for researchers evaluating passive smoking exposure in indoor settings, especially in domestic environments. Since numerous countries have introduced smoking bans in enclosed public places, domestic environments have become the main sources of passive smoking exposure (World Health Organization 2007). We believe researchers should determine the independent contributions of secondhand and thirdhand smoke when they assess the magnitude of pollutant intake due to passive smoking exposure.
